# Impact of a Ketogenic Diet on Metabolic Parameters in Patients with Obesity or Overweight and with or without Type 2 Diabetes: A Meta-Analysis of Randomized Controlled Trials

**DOI:** 10.3390/nu12072005

**Published:** 2020-07-06

**Authors:** Yeo Jin Choi, Sang-Min Jeon, Sooyoung Shin

**Affiliations:** 1 Clinical Trial Center, Hallym University Hospital, Anyang 14066, Korea; utds811@hotmail.com; 2 College of Pharmacy, Ajou University, Suwon 16499, Korea; smjeon@ajou.ac.kr; 3 Research Institute of Pharmaceutical Science and Technology (RIPST), Ajou University, Suwon 16499, Korea

**Keywords:** ketogenic diet, glycemic control, obesity, type 2 diabetes

## Abstract

The aim of this meta-analysis was to explore the efficacy of a ketogenic diet in metabolic control in patients with overweight or obesity and with or without type 2 diabetes. Embase, PubMed, and Cochrane Library were searched for randomized controlled trials that enrolled patients with overweight or obesity on a ketogenic diet for metabolic control. Fourteen studies were included in meta-analysis. The effects of ketogenic diets on glycemic control were greater for diabetic patients relative to those of low-fat diets, indicated by lower glycated hemoglobin (SMD, −0.62; *p* < 0.001) and homeostatic model assessment index (SMD, −0.29; *p* = 0.02), while comparable effects were observed for nondiabetic patients. Ketogenic diets led to substantial weight reduction (SMD, −0.46; *p* = 0.04) irrespective of patients’ diabetes status at baseline and improved lipid profiles in terms of lower triglyceride (SMD, −0.45; *p* = 0.01) and greater high-density lipoprotein (SMD, 0.31; *p* = 0.005) for diabetic patients. Other risk markers showed no substantial between-group difference post intervention. Our study findings confirmed that ketogenic diets were more effective in improving metabolic parameters associated with glycemic, weight, and lipid controls in patients with overweight or obesity, especially those with preexisting diabetes, as compared to low-fat diets. This effect may contribute to improvements in metabolic dysfunction-related morbidity and mortality in these patient populations.

## 1. Introduction

Obesity rates worldwide have tripled since the 1970s, and with the increasing prevalence and severity, obesity plays as a major underlying risk factor for chronic disorders, such as type 2 diabetes mellitus (T2DM), cardiovascular disorders, metabolic syndromes, chronic renal disorders, and malignancies, consequently increasing morbidity and mortality [1]. Each year, the number of population with obesity is constantly growing, with approximately 38% and 20% of world’s adult population to be overweight and obese by 2030, respectively, and the proportion of population with overweight and obesity is anticipated to be even higher at approximately over 85% in the United States [2]. Besides, the escalating incidence of chronic disorders secondary to childhood obesity stresses the importance of dietary modifications in the population worldwide [3]. 

The current treatment guidelines for obesity recommend dietary restriction, specifically caloric restriction, as the first nutritional modification for weight loss [4]. Many patients with obesity start with a low-fat diet, which is composed of a relatively large amount of carbohydrates, because fats contain the highest calories among macronutrients [5,6]. A ketogenic diet, on the other hand, is a high-fat, low-carbohydrate diet, limiting carbohydrate intake to 5% to 10% of total daily dietary requirements and replacing the remaining with dietary fat and adequate protein (1g/kg) [7]. Ketogenic diets have been recommended as nonpharmacological treatment for medication-refractory epilepsy in children and adults since 1921 [8,9]. The evident anticonvulsive mechanism of the ketogenic diet still needs to be elucidated [7,10]. Nevertheless, ketogenic diets have shown to reduce seizure frequency in patients with medication-refractory epilepsy, and some patients even reached complete and sustained remission [10]. Moreover, ketogenic diets were indeed the principal therapeutic option for diabetes before the discovery of insulin in the early 20th century [11,12]. Dr. Elliot Joslin’s Diabetic Diet, for example, consisted of 75% of energy from fat, 20% from protein, and only 5% from carbohydrate [13], which was also supported by Dr. Frederick Allen, a physician who advocated a low-carbohydrate approach for diabetic patients [14]. However, the demand for the strict dietary option had been declined with the use of insulin as a therapeutic option for diabetes. 

In recent years, researchers have investigated the benefits of ketogenic diets in patients with obesity. Its mechanism for weight loss is mainly associated with reducing insulin levels, which promotes redirecting lipid metabolism from synthesis and storage toward breakdown and oxidation, consequently inducing nutritional ketosis, mimicking metabolic starvation in the body to utilize ketone bodies as alternative energy sources [15]. According to Dashti et al. [16], a 24-week ketogenic diet in patients with obesity with body mass index (BMI) greater than 35 kg/m^2^ not only decreased body weight and BMI of the patients but also significantly improved glycemic and lipid profiles, as indicated by a notable reduction in blood glucose, triglyceride, and low-density lipoprotein (LDL) cholesterol along with elevation of high-density lipoprotein (HDL) cholesterol; nonetheless, the appropriateness of ketogenic diets in patients with obesity is still not firmly established. Therefore, this study aims to investigate the clinical effects of ketogenic diets in patients with overweight or obesity and with or without T2DM as compared to low-fat diets by assessing changes in metabolic parameters related to glycemic, weight, and lipid controls, along with other cardiovascular and renal risk markers. 

## 2. Materials and Methods

The Preferred Reporting Items for Systematic Reviews and Meta-analyses (PRISMA) was used to guide our methodology and to prepare this article [17,18]. 

### 2.1. Data Sources

A systematic literature search of Embase, PubMed, and Cochrane Library was performed to identify randomized controlled trials (RCTs) (published until 11 February 2020) of patients with overweight or obesity and with or without T2DM that evaluated the effects of a ketogenic diet on glycemic control, weight loss, and lipid profile as primary endpoints. The initial database search strategies were pre-specified as follows: the search term was composed of a combination of key words and Medical Subject Headings (ketogenic diet or high-fat diet) in title/abstract AND (glucose or glycated hemoglobin (HbA1c) in title/abstract; search filters were set as “clinical trials” and “humans.” Reference lists of previous reviews were also screened to identify eligible RCTs. Ethics committee review and informed consent from study participants were waived as this meta-analysis was conducted by pooling existing patient data extracted from published primary research. 

### 2.2. Study Selection and Data Collection

Two reviewers (Shin and Choi) independently searched the databases for relevant RCTs. Those studies identified through the initial search were screened for eligibility using the following inclusion criteria: (1) RCTs of the metabolic effects of ketogenic diets in patients with overweight or obesity, (2) glycemic outcomes must be associated with a ketogenic diet but regardless of patients’ T2DM status at baseline, (3) sufficient raw data obtainable from primary research, and (4) studies published in English. No age restrictions were applied in the selection of relevant studies. Review articles, case reports, and duplicate studies were excluded from our meta-analysis. Study attributes, including first author and year of publication; country; trial design; intervention duration; comparator diets; number of patients; patient age and sex; fasting glucose (FG); BMI and T2DM status at baseline; and detailed inclusion criteria regarding age, BMI, and glycated hemoglobin (HbA1c) parameters, were extracted independently by the two reviewers utilizing a predefined summary format. Included studies had randomized patients to either a ketogenic diet or a control diet and reported pre-post glycemic indices, such as FG and HbA1c, along with other metabolic parameters related to weight and lipid controls. In case of multiple intervention periods followed up in a single study, the intervention duration in the present meta-analysis was defined as the last available study period in each study. Any controversial issues and disagreements were resolved by consensus between authors. 

### 2.3. Risk of Bias and Quality Assessment of Evidence

The quality assessment of the included RCTs was carried out per the Cochrane Risk of Bias approach on the basis of the following accounts: randomization adequacy, allocation concealment, blinding of participants, investigators and outcome assessors, follow-up duration and drop-out rates, and application of intention-to-treat analysis. Studies were then scored as low, unclear, or high risk of bias in the following domains: selection, performance, detection, attrition, reporting, and other bias. Additionally, we rated the quality of each evidence as high, moderate, low, and very low in effect estimates for individual outcomes, based on the Grading of Recommendations, Assessment, Development and Evaluation (GRADE) approach, in which studies are evaluated on five aspects: precision, consistency of effect, directness, risk of bias, and publication bias [19,20]. Disagreement on risk of bias and quality of evidence was resolved through discussion among authors.

### 2.4. Data Analysis and Statistical Methods

The efficacy of a ketogenic diet was evaluated by pooling data from individual RCTs, and the overall effect size of continuous variables were presented as the weighted standardized mean difference (SMD) with 95% confidence intervals (CIs). Post-treatment laboratory values in the ketogenic diet-receiving patients were compared against those in comparator group patients. The primary serum levels assessed in this meta-analysis included FG, HbA1c, fasting insulin, C-peptide, total cholesterol, triglyceride, LDL, and HDL, along with other laboratory findings, such as C-reactive protein (CRP) and serum creatinine (SCr). Data on the homeostatic model assessment (HOMA) index of insulin resistance, patient weight, BMI, waist circumference, systolic blood pressure (SBP), and diastolic blood pressure (DBP) were also collected. Heterogeneity across the studies was quantitatively evaluated by Q-statistic and *I*^2^ index. When *I*^2^ < 50%, which was considered as a low heterogeneity, a Mantel–Haenszel fixed-effects model was implemented; otherwise (*I*^2^ > 50%), a random-effects model was adopted. When the mean and standard deviation were not specifically given, we calculated those summary statistics by utilizing other effect estimates and measures of dispersion. In consideration of T2DM status at baseline and to minimize potential sources of heterogeneity, subgroup analyses were designed when appropriate in accordance with the presence of T2DM as comorbidity among included patients. *p*-values were estimated from 2-sided tests and considered statistically significant when below 0.05. Statistical analysis was conducted using RevMan Version 5.3 (The Cochrane Collaboration). Begg’s funnel plots and Egger’s linear regression tests were performed to detect the existence of publication bias; a symmetric funnel plot suggests a low risk of publication bias. 

## 3. Results

### 3.1. Selection of Relevant Studies

**Figure 1:**
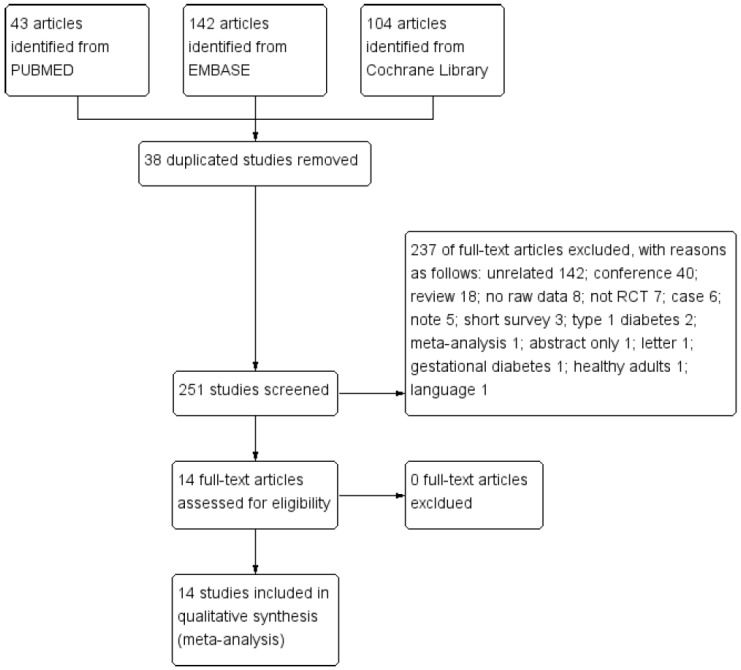
Study selection diagram. Abbreviation: RCT, randomized controlled trial.

The process of searching and identifying relevant studies is presented as Figure 1. The initial database search yielded 289 citations (43 publications from PubMed, 142 from Embase, and 104 from Cochrane Library), of which 38 duplicates were excluded. The remaining 251 trials underwent further screening of titles and abstracts. Resultantly, a total of 237 articles were excluded due to the following reasons: unrelated 142, conference abstracts 40, reviews 18, no raw data available 8, not RCTs 7, case studies 6, notes 5, short surveys 3, type 1 diabetes 2, meta-analysis 1, abstract only 1, letter 1, gestational diabetes 1, healthy adults 1, and not English 1. After full-text review, 14 RCTs met the inclusion criteria for quantitative analysis, which enrolled a total of 734 participants with overweight or obesity, including 444 diabetic patients and 290 non-diabetic patients. 

### 3.2. Study Characteristics

The characteristics of eligible RCTs are summarized in Table 1. The comparator group patients received varying types of low-fat diets, such as low-calorie diet, low-fat diet, low-fat low-glycemic index diet, high-carbohydrate low-fat diet, moderate-carbohydrate calorie-restricted low-fat diet, etc. 

**Figure 2:**
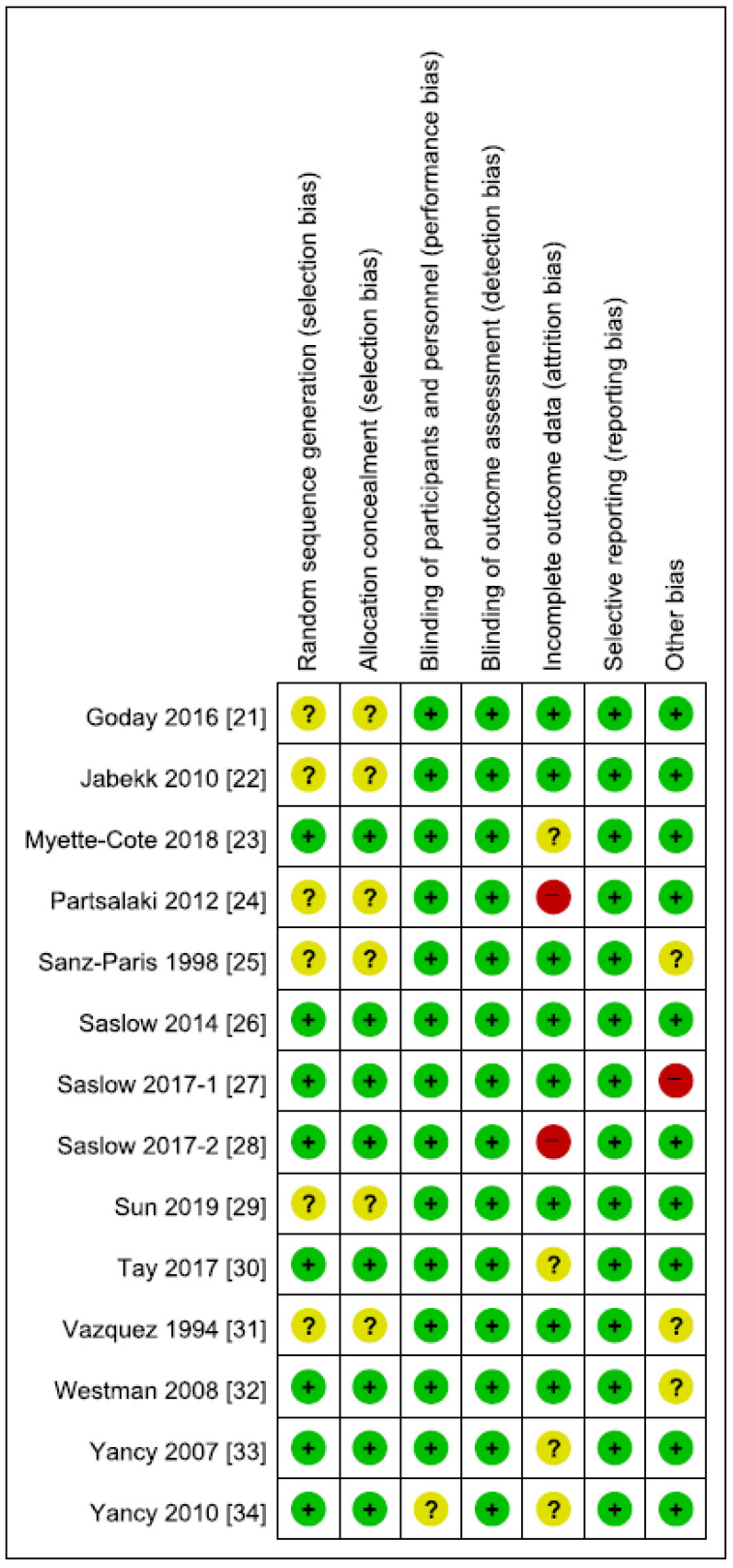
Risk of bias summary.

In terms of inclusion criteria regarding baseline comorbidity, 8 studies enrolled T2DM patients [21,23,25,26,27,28,30,32] (in 2 studies prediabetic patients were also included) [26,27], whereas the remaining 6 studies incorporated non-diabetic patients [22,24,29,31,33,34]. Seven trials were conducted in the United States [26,27,28,31,32,33,34]; 2 in Spain [21,25]; and 1 in Norway [22], Canada [23], Greece [24], China [29], and Australia [30] each. There was no significant between-group difference in serum levels of FG at pretreatment state in each study, with the mean value ranging from 84.3 to 185 mg/dL in ketogenic diet groups and from 80.2 to 170 mg/dL in comparator groups. The inclusion criteria with respect to age, BMI, or HgA1c at baseline appeared varied in each study, which are summarized in Table 1. One study included female patients only [29]. Adverse effects were reported in only one study [21]. Individual RCT appraisal on each risk of bias is reported in Figure 2. Two of the studies had a high risk of attrition bias [24,28], and one had a high risk of bias due to imbalance in baseline triglyceride levels between groups [27]. Otherwise, the risk of bias assessment of the included studies was generally acceptable, and the funnel plots did not signify any evidence of publication bias.

### 3.3. The Effects of a Ketogenic Diet on Glycemic Control 

**Figure 3:**
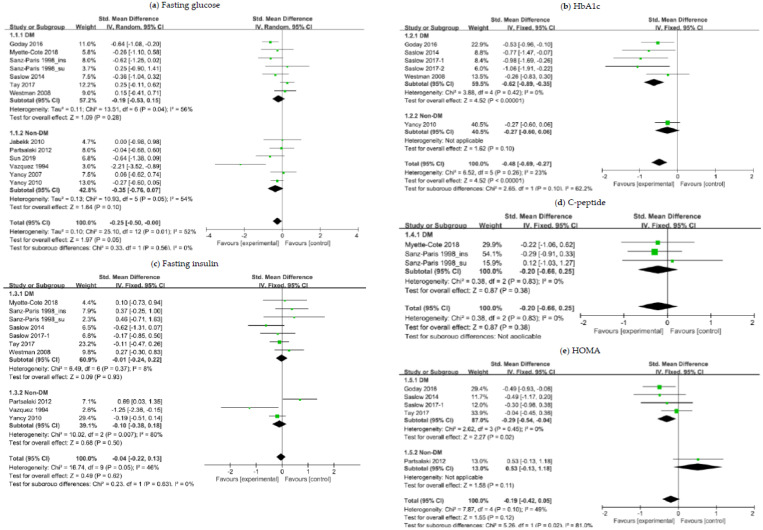
Forest plots for association between glycemic control and ketogenic diet in patients with overweight or obesity and with or without T2DM: (**a**) Changes in fasting glucose; (**b**) changes in HbA1c; (**c**) changes in fasting insulin; (**d**) changes in C-peptide; (**e**) changes in HOMA. Abbreviations: CI, confidence interval; DM, diabetes mellitus; Non-DM, non-diabetes mellitus; HbA1c, glycated hemoglobin; HOMA, homeostatic model assessment; T2DM, type 2 diabetes mellitus.

We stratified participants in accordance with the T2DM status at baseline. Subgroup analysis of patients with overweight or obesity and with preexisting T2DM revealed a substantial difference in the change of HbA1c (SMD, −0.62; 95% CI, −0.89 to −0.35; *I*^2^ = 0%; moderate quality evidence) and HOMA (SMD, −0.29; 95% CI, −0.54 to −0.04; *I*^2^ = 0%; moderate quality evidence) (Figure 3). Subgroup analysis of nondiabetic patients showed no significant decrease in the glycemic index in ketogenic diet groups relative to comparator groups. In overall patients, the ketogenic diet appeared more effective in lowering FG (SMD, −0.25; 95% CI, −0.50 to −0.00; *I*^2^ = 52%; low quality evidence) and HbA1c (SMD, −0.48; 95% CI, −0.69 to −0.27; *I*^2^ = 23%; moderate quality evidence) than comparator diets (Figure 3). The mean between-group difference in HbA1c changes was −0.5% (*p* < 0.001) and −0.42% (*p* < 0.001) in diabetic patients and overall patients, respectively. 

In consideration of potential confounding effects owing to heterogeneity across RCTs, we performed an additional analysis after excluding two RCTs [23,25] from meta-analysis for relatively short-term follow-up [23,25] and antihyperglycemic use on the day of serum sampling for glucose and insulin measurement [25]. Similar results were obtained regarding the effect of a ketogenic diet on fasting insulin, whereas its effect on FG in overall patients was no longer statistically significant (*p* = 0.10). More detailed information about these analysis results can be found in Supplementary Material (Figure S1).

### 3.4. The Effects of a Ketogenic Diet on Weight Control

**Figure 4:**
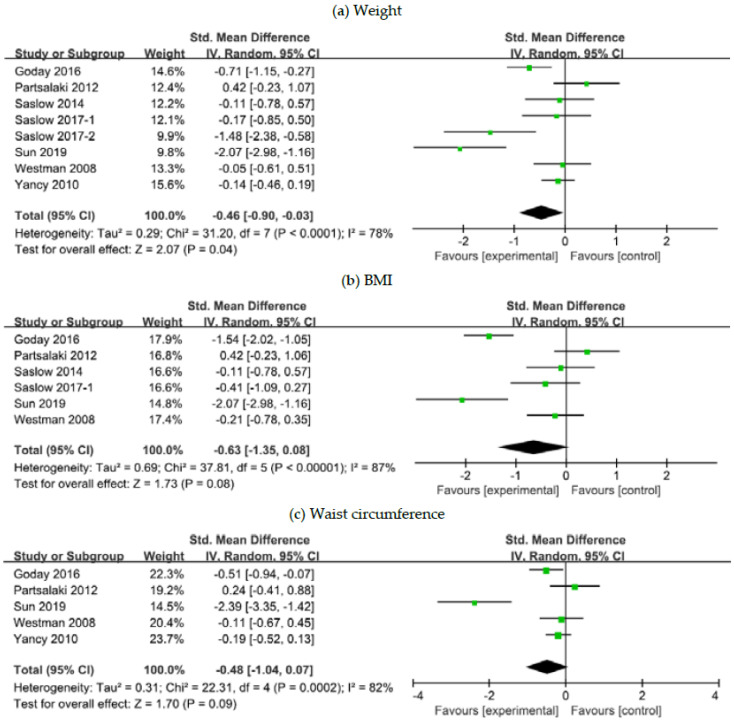
Forest plots for association between weight control and ketogenic diet in patients with overweight or obesity and with or without T2DM: (**a**) Changes in body weight; (**b**) changes in BMI; (**c**) changes in waist circumference. Abbreviations: CI, confidence interval; BMI, body mass index; T2DM, type 2 diabetes mellitus.

Eight studies reported changes in weight-related parameters post the dietary intervention [21,24,26,27,28,29,32,34]. Meta-analysis demonstrated that ketogenic diet patients were more likely to experience a greater weight reduction compared to those on comparator diets (SMD, −0.46; 95% CI, −0.90 to −0.03; *I*^2^ = 78%; moderate to low quality evidence) (Figure 4). The mean between-group difference in weight changes was −7.78 kg (*p* < 0.001) in diabetic patients and −3.81 kg (*p* = 0.01) in overall patients. The SMD in BMI and waist circumference reduction was not associated with statistical significance but approached borderline significance in favor of the ketogenic diet groups: *p* = 0.08 and *p* = 0.09, respectively (Figure 4). 

### 3.5. The Effects of a Ketogenic Diet on Lipid Panel

**Figure 5:**
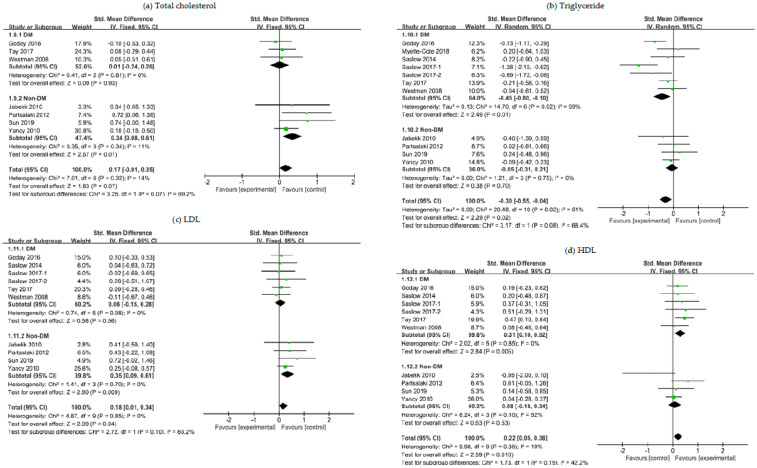
Forest plots for association between lipid control and ketogenic diet in patients with overweight or obesity and with or without T2DM: (**a**) Changes in total cholesterol; (**b**) changes in triglyceride; (**c**) changes in LDL; (**d**) changes in HDL. Abbreviations: CI, confidence interval; DM, diabetes mellitus; Non-DM, non-diabetes mellitus; LDL, low-density lipoprotein; HDL, high-density lipoprotein; T2DM, type 2 diabetes mellitus.

Study patients were stratified in accordance with the T2DM status at baseline. Subgroup analysis of patients with overweight or obesity and with T2DM revealed that the ketogenic diet showed a greater efficacy in controlling serum levels of triglyceride (SMD, −0.45; 95% CI, −0.80 to −0.10; *I*^2^ = 59%; moderate quality evidence) while raising HDL levels (SMD, 0.31; 95% CI, 0.10 to 0.52; *I*^2^ = 0%; moderate quality evidence) than comparator diets (Figure 5). Meanwhile, among nondiabetic patients, a relatively higher increase in total cholesterol (SMD, 0.34; 95% CI, 0.08 to 0.61; *I*^2^ = 11%; moderate to low quality evidence) and in LDL (SMD, 0.35; 95% CI, 0.09 to 0.61; *I*^2^ = 0%; moderate to low quality evidence) was observed with the ketogenic diet (Figure 5). When all patients were included in the analysis regardless of T2DM status, the ketogenic diet was associated with a greater reduction in triglyceride levels (*p* = 0.02; moderate to low quality evidence) and an increase in serum levels of both LDL (*p* = 0.04; moderate to low quality evidence) and HDL (*p* = 0.01). 

The mean between-group difference in triglyceride changes was −35.12 mg/dL (*p* = 0.002) in diabetic patients and −20.65 mg/dL (*p* = 0.02) in overall patients. The mean difference in HDL changes was +3.48 mg/dL (*p* = 0.003) in diabetic patients and +1.91 mg/dL (*p* = 0.02) in overall patients. Meanwhile, the mean difference in total cholesterol changes was +10.13 mg/dL (*p* = 0.01) in nondiabetic patients and +6.82 mg/dL (*p* = 0.03) in overall patients. The mean difference in LDL changes was +9.2 mg/dL (*p* = 0.009) in nondiabetic patients and +5.89 mg/dL (*p* = 0.02) in overall patients. 

In order to remove a potential confounding factor, we repeated meta-analysis after excluding the RCT of nondiabetic patients by Yancy et al. (2010) [34], where the control arm patients received orlistat, a weight loss therapy that functions by preventing dietary fat from being absorbed into the body, as an add-on to a low-fat diet, which might have affected post-intervention lipid results. This separate analysis showed that the overall effect estimate trends and statistical significance did not substantially changed and that in nondiabetic patients the ketogenic diet was again more likely to increase total cholesterol (SMD, 0.65; 95% CI, 0.21 to 1.09; *I*^2^ = 0%; moderate to low quality evidence) and LDL (SMD, 0.53; 95% CI, 0.09 to 0.96; *I*^2^ = 0%; moderate to low quality evidence). 

Additionally, similar results were confirmed in a separate analysis where a potential confounding effect due to heterogeneity in terms of the length of follow-up was eliminated by excluding one more RCT of diabetic patients [23] from meta-analysis of triglyceride control: The effect of a ketogenic diet on triglyceride changes in both T2DM patients and overall patients remained statistically significant (*p* = 0.005 and *p* = 0.02, respectively). More detailed information about this additional analysis can be found in Supplementary Material (Figure S2).

### 3.6. The Effects of a Ketogenic Diet on Other Risk Markers

**Figure 6:**
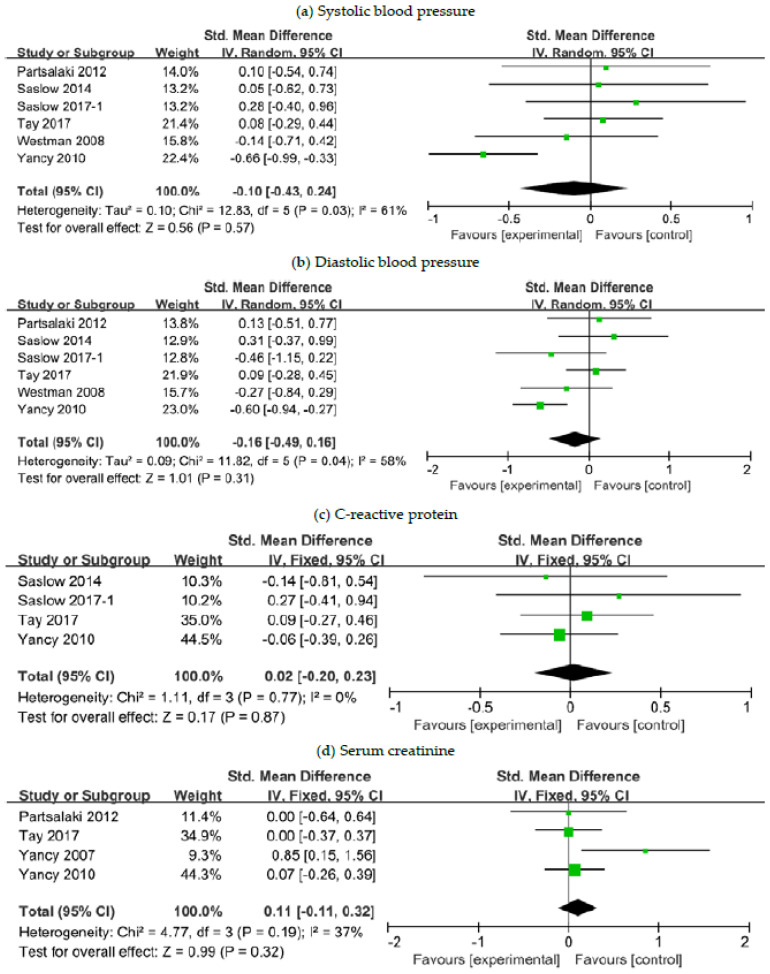
Forest plots for association between other risk markers and ketogenic diet in patients with overweight or obesity and with or without T2DM: (**a**) Changes in systolic blood pressure; (**b**) changes in diastolic blood pressure; (**c**) changes in C-reactive protein; (**d**) changes in serum creatinine. Abbreviations: CI, confidence interval; T2DM, type 2 diabetes mellitus.

We combined data for other cardiovascular and renal risk markers, including SBP, DBP, CRP and SCr, reported in six studies [24,26,27,30,32,34]. Additional meta-analysis was performed to evaluate the safety of the ketogenic diet in terms of these parameters relative to comparator diets in patients with overweight or obesity. Our analysis results revealed that there was no substantial between-group difference in post-intervention changes in all of the aforementioned parameters (Figure 6).

## 4. Discussion

In this meta-analysis of 14 RCTs, we investigated the efficacy of a ketogenic diet relative to comparator diets (primarily low-fat diets) on the control of metabolic parameters in patients with overweight or obesity and with or without preexisting T2DM. The main findings included: (1) a ketogenic diet for 3 to 12 months was more effective for glycemic control as indicated by a significant reduction in HbA1c and HOMA values for diabetic patients. Notably, the mean post-intervention improvement in HbA1c levels was −0.5% (*p* < 0.001) and −0.42% (*p* < 0.001) in diabetic patients and overall patients, respectively, indicating clinically relevant improvement in glycemic control in these patient populations. (2) A ketogenic diet for 4 weeks to 12 months was linked to a greater weight loss in patients with overweight or obesity regardless of T2DM. The mean post-intervention weight change was −7.78 kg (*p* < 0.001) and −3.81 kg (*p* = 0.01) in diabetic patients and overall patients, respectively. (3) A ketogenic diet for 4 days and up to 2 years led to improved lipid profiles for diabetic patients, such as lower triglyceride and higher HDL levels, whereas for nondiabetic patients an increase in total cholesterol and LDL levels. The mean post-intervention improvement in triglyceride levels was −35.12 mg/dL (*p* = 0.002) and −20.65 mg/dL (*p* = 0.02) in overall patients. (4) The effect of a ketogenic diet on cardiovascular and renal risk markers was comparable to that of a low-fat diet. The findings of this meta-analysis demonstrated that a ketogenic diet can be a more beneficial dietary option for diabetic patients with overweight or obesity and to improve metabolic factors related to glycemic, weight, and lipid controls. 

The incidences of chronic disorders including T2DM, cardiovascular disorders, renal disorders and malignancies rise with increasing life expectancy, and the majority of these disorders are closely associated with excessive nutrients or obesity [2]. Thus, the importance of a proper diet cannot be neglected in chronic disease prevention and management, and various dietary plans including a conventional diet for weight loss are currently available for weight loss or prevention of obesity-associated disorders [35]. Recent studies [36,37] suggested that caloric restriction without malnutrition, the cornerstone of weight loss, may help to extend lifespan by preventing age-associated pathogenesis. Besides, inducing starvation via dietary regimens of alternate-day fasting [38] or intermittent fasting [39] has shown to reduce caloric intake and improve health markers related to cardiovascular risk in populations irrespective of obesity status. 

A ketogenic diet, another method to induce metabolic starvation in the body, strictly limits the intake of carbohydrates, the major sources for glucose, and increases fat consumption instead [40]. This high-fat diet is currently recommended as a treatment for patients with medication-refractory epilepsy [8,9]. The exact mechanism of its anticonvulsive activity is still under debate; however, dietary fatty acids, including omega-6 and omega-3 long chain polyunsaturated fatty acids, have shown to regulate signal-transduction pathways in the brain [41]. Moreover, the neuroprotective effects of the ketogenic diet have also been reported in studies, which would benefit patients with Alzheimer’s or Parkinson’s disease [42,43]. Recently, some studies advocated the beneficial effects of the ketogenic diet in weight management and prevention of metabolic syndrome by inducing nutritional ketosis [15]. Carbohydrate restriction to less than 50 g per day triggers gluconeogenesis and ketogenesis in the liver, production of ketone bodies such as acetoacetic acid and beta-hydroxybutyric acid from fatty acids in replacement of glucose [44]. Ketogenesis usually takes place once endogenous glucose production is depleted and lowers insulin levels in the blood, which further limits the storage of fat and glucose in the body [44]. 

In accordance with the biochemical hypothesis, our meta-analysis evaluating the effects of a ketogenic diet over low-fat dietary plans demonstrated significantly greater weight loss in patients with overweight or obesity. BMI and waist circumference, on the other hand, were shown to have a decreasing tendency with a ketogenic diet, albeit not statistically significant. According to Johnston et al. [45], 4 weeks of a ketogenic diet was sufficient for patients with obesity to decrease food intake by regulating appetite and hunger and subsequently lose 6.34 kg on average. Interestingly, a previous meta-analysis [46] displayed much more substantial weight loss with a long-term very-low carbohydrate ketogenic diet when compared with a low-fat diet, a conventional diet for weight loss, but the appropriateness of a long-term ketogenic diet for weight loss should be thoroughly evaluated before being endorsed as a viable treatment option for obesity. 

In this study, the benefits of a ketogenic diet were considerably greater in diabetic patients with obesity and, reflected by markedly lower HbA1C levels. The primary reasons for improved glycemic profiles are involved with lowered postprandial glucose and enhanced insulin sensitivity. Although our study did not evaluate the changes of postprandial glucose levels with a ketogenic diet, we could assume that carbohydrate restriction from a ketogenic diet decreased postprandial glucose in diabetic patients since postprandial glucose regulation is closely related to the amount of carbohydrates consumed [47]. On the contrary, enhanced insulin sensitivity with a ketogenic diet in diabetic patients with obesity was confirmed in our analysis, as supported by significantly reduced HOMA values irrespective of insignificant changes in fasting insulin and C-peptide levels, both of which are markers for insulin secretion. Obesity induces insulin resistance, mediated by chronic low-grade inflammation including endoplasmic reticulum (ER) stress, mitochondrial dysfunction, and hyperinsulinemia secondary to diminished insulin excretion via the kidney and the liver along with increased insulin production resulting from the increased number of pancreatic beta-cells, and weight loss is reported to attenuate or reverse obesity-induced insulin resistance [48,49]. Although our study results did not provide evidence on direct association between insulin sensitivity and weight loss, enhanced insulin sensitivity from a ketogenic diet can be expected in the context of biological responses to weight loss, and such a relationship has been demonstrated in a previous meta-analysis that reported substantial weight loss-dependent glycemic improvement with a low-carbohydrate diet [50]. 

In 2019, American Diabetes Association (ADA) newly endorsed low-carbohydrate diet as part of medical nutrition therapy options in diabetic patients based on the study results denoting enhanced glycemic control with a very low-carbohydrate diet inducing nutritional ketosis in T2DM patients [27,50,51,52]. Besides, nutritional ketosis has shown the capacity to reduce the use of hypoglycemic agents in diabetic patients [32,52]. However, ADA highly stresses the importance of individualized dietary plans based on personal eating patterns, preferences and metabolic goals because a low-carbohydrate high-fat diet may have some issues with long-term sustainability [51]. According to a meta-analysis performed by Sainsbury et al. [50], the enhanced effect of carbohydrate-restriction on glycemic control is most significant during the first 3 to 6 months and becomes less effective thereafter. Our meta-analysis for glycemic control incorporated 4 studies [27,28,30,34] that had intervention duration of at least 8 months or longer, and hence, further study on long-term sustainability along with long-term benefits of a ketogenic diet in glycemic control is warranted. 

Obesity has a clear correlation with the incidence of cardiovascular disorders including hypertension, coronary artery disease, heart failure and sudden death, and many patients with insulin resistance, the main etiology of T2DM, tend to have high triglyceride and low HDL levels [53,54,55]. Therefore, we analyzed the impact of a ketogenic diet on lipid controls and cardiovascular risk markers and found similar results to previous studies [46,56,57] that evaluated the effects of a low-carbohydrate diet; our analysis revealed that a ketogenic diet decreased triglycerides while increasing total cholesterol secondary to increased LDL and HDL levels in patients with overweight or obesity, but no significant changes in other cardiovascular risk markers including blood pressure, CRP, and SCr were detected. Considering low HDL and high triglyceride levels being independent risk factors for insulin resistance and cardiovascular disorders [58], our analysis suggested that a ketogenic diet has cardioprotective effects in diabetic patients as the effects of a ketogenic diet on triglyceride and HDL were much more significant in diabetic patients. However, cardioprotective effects of a ketogenic diet in patients with obesity but no T2DM are yet to be determined due to relatively higher post-intervention levels of LDL and total cholesterol. Interestingly, the study conducted by Westman et al. [59] showed that a ketogenic diet changed the composition of LDL subclasses, increasing the proportion of large-sized buoyant LDL with cardiovascular protective effects while significantly decreasing small-sized dense LDL, which is the primary cause of atherogenesis in the arterial intima. Thus, it would be important to determine whether increased LDL levels observed in patients with obesity but no T2DM in our study are due to the increase of large-sized buoyant LDL, which can potentially contribute to the cardioprotective effects by ketogenic diets. Some prospective studies registered on the ClinicalTrials.gov are currently ongoing to analyze the effects of ketogenic diets on the ratio between triglyceride and HDL, and on the composition of LDL subclasses with its long-term clinical outcomes in cardiovascular diseases.

To the best of our knowledge, this is the first study to perform meta-analysis with RCTs to investigate the overall impact of ketogenic diets on glycemic control, weight loss, lipid control and cardiovascular and renal risk markers over varied low-fat dietary plans including low-calorie or high-carbohydrate diets. Based on our study results, patients with overweight or obesity and with underlying T2DM are more likely to receive benefits in terms of weight loss, improvement of glycemic and lipid controls from ketogenic diets. Additionally, the clinical significance of a ketogenic diet in cancer patients is assessed in recent studies because diabetes-specific factors including insulin resistance, hyperglycemia, and hyperinsulinemia, which can be reversed by a ketogenic diet as shown in our study, play as biological links to site-specific malignancies, especially in breast, lung, kidney, and pancreas [60,61,62]. In light of numerous study results, a ketogenic diet can provide promising effects in many disorders including obesity, cardiovascular disorders, T2DM, and possibly cancer. Some questions, nonetheless, still remained to be answered before justifying the appropriateness of ketogenic diets as obesity treatment: long-term sustainability, safety, and clinical significance of cardioprotective and anticancer activity. 

Our study results confirmed the efficacy of a ketogenic diet on improving metabolic control in patients with overweight or obesity, especially those with underlying T2DM, nevertheless some limitations need to be acknowledged. Meta-analysis was performed incorporating RCTs with different study design and control diets, albeit mostly low-fat based diets. Varying intervention durations were adopted in included studies. Some subgroup analysis may have been underpowered to detect a difference between groups owing to small sample size. Surrogate markers were used to explore the metabolic efficacy of a ketogenic diet, such as serum levels of FG, HbA1c, and lipid components, instead of evaluating more patient-important endpoints, such as cardiovascular and renal events, and other obesity-related morbidity and mortality. However, the findings from this meta-analysis with respect to the impact of a ketogenic diet on those surrogate markers can validate and provide insight into the effects of a ketogenic diet on the more clinically important endpoints. 

## 5. Conclusions

A ketogenic diet was more effective in improving weight control and metabolic parameters related to glycemic and lipid controls in patients with overweight or obesity, especially those with preexisting T2DM, as compared to low-fat based comparator diets. Its effects on other risk markers, such as blood pressure, CRP and SCr were comparable to those of low-fat diets. Further studies are warranted to determine the long-term sustainability of a ketogenic diet and its effects on the more clinically important endpoints such as obesity-related morbidity and mortality.

## Figures and Tables

**Table 1:** Characteristics of included studies.

Study (Country)	Trial Design	Intervention Duration	Diet	N	Age, Mean ± SD (Years)	Female, N (%)	FG, Mean ± SD (mg/dL)	BMI, Mean ± SD (kg/m^2^)	Baseline T2DM	Inclusion Criteria
Goday 2016 [21] (Spain)	Prospective, open-label, multi-centric, parallel group randomized controlled trial	4 months	I: very low-calorie-ketogenic diet	45	54.89 ± 8.81	30 (66.6)	136.87 ± 34.43	33.25 ± 1.52	Yes	Age: 30–65 years; BMI: 30–35 kg/m^2^
			C: low-calorie diet	44	54.17 ± 7.97	28 (63.6)	142.81 ± 44.26	32.88 ± 1.60		
Jabekk 2010 [22] (Norway)	Randomized controlled trial	10 weeks	I: low carbohydrate, ketogenic diet	8	-	8 (100)	88.2 ± 5.4	32.9 ± 4.5	No	Age: 20–40 years; BMI: ≥25 kg/m^2^
			C: regular diet	8	-	8 (100)	90.0 ± 5.4	31.7 ± 4.2		
Myette-Cote 2018 [23] (Canada)	Randomized controlled crossover trial	4 days	I: low-carbohydrate high-fat diet	11	64 ± 8	7 (63.6)	151.2 ± 34.2	34.0 ± 8.0	Yes	HbA1c >6.5% or FG >7.0 mmol/L or 2-h OGTT >11.1 mmol/L
			C: low-fat low-glycemic index guidelines diet				149.4 ± 37.8			
Partsalaki 2012 [24] (Greece)	Randomized controlled trial	6 months	I: ketogenic diet	21	12.8 ± 2.1	11 (52.4)	84.3 ± 9.8	30.0 ± 4.3	No	Age: 8–18 years; BMI: >95th percentile for gender and age
			C: hypocaloric diet	17	12.7 ± 2.8	10 (58.8)	80.2 ± 17.6	28.1 ± 3.1		
Sanz-Paris 1998 [25] (Spain)	Randomized controlled trial	120 min	I: low-carbohydrate, high-fat formulation	27	65 ± 7	33 (64)	185 ± 56	29.2 ± 2.7	Yes	Age: 45–72 years; good glycemic control (mean HbA1c: 6.5 ± 0.9%)
			C: high-carbohydrate, low-fat formulation	25			170 ± 48			
Saslow 2014 [26] (US)	Single site, parallel group, balanced randomization (1:1) trial	3 months	I: low-carbohydrate ketogenic diet	16	64.8 ± 7.7	9 (56.3)	124.4 ± 28.3	36.2 ± 8.2	Yes(including prediabetes)	Age: ≥18 years;BMI: ≥25 kg/m^2^; HbA1c: ≥6.5% (T2DM) or >6% (prediabetes)
			C: moderate-carbohydrate, calorie-restricted, low-fat diet	18	55.1 ± 13.5	16 (88.9)	140.6 ± 34.3	37.4 ± 6.4		
Saslow 2017-1 [27] (US)	Single site, parallel group, balanced randomization (1:1) trial	12 months	I: low-carbohydrate ketogenic diet	16	64.8 ± 7.7	9 (56.3)	-	36.2 ± 8.2	Yes(including prediabetes)	Age: ≥18 years; BMI: ≥25 kg/m^2^; HbA1c: ≥6.5% (T2DM) or >6% (prediabetes)
			C: moderate-carbohydrate, calorie-restricted, low-fat diet	18	55.1 ± 13.5	16 (88.9)	-	37.4 ± 6.4		
Saslow 2017-2 [28] (US)	Parallel-group, balanced randomization (1:1) trial	32 weeks	I: very low-carbohydrate ketogenic diet	12	53.0 ± 10.2	6 (50)	-	-	Yes	Age: ≥18 years; BMI: ≥25 kg/m^2^; HbA1c: ≥6.5–9%
			C: American Diabetes Associations’ “Create Your Plate” diet	13	58.2 ± 6.7	9 (69)	-	-		
Sun 2019 [29] (China)	Randomized controlled trial	4 weeks	I: low-carbohydrate, ketogenic diet	18	20.9 ± 3.7	18 (100)	84.6 ± 7.2	25.0 ± 2.9	No	Age: 18–30 years; BMI: ≥23 kg/m^2^; past 6-month weight variation: <2kg; sedentary lifestyle
			C: normal diet	17	21.6 ± 3.9	17 (100)	82.8 ± 9.0	24.8 ± 3.2		
Tay 2017 [30] (Australia)	Single-center, parallel-groups, randomized controlled trial	2 years	I: low-carbohydrate, high-unsaturated/low-saturated fat diet	58	58 ± 7.6	21 (36)	140.4 ± 37.8	34.2 ± 4.2	Yes	Age: 35–68 years; BMI: 26–45 kg/m^2^; HbA1c: 7.0% or antidiabetic treatment
			C: high-carbohydrate, low-fat diet	57	58 ± 7.5	28 (49)	151.2 ± 41.4	35.1 ± 4.1		
Vazquez 1994 [31] (US)	Randomized controlled trial	28 days	I: ketogenic diet	8	48 ± 3	8 (100)	86.4 ± 5.4	41 ± 5	No	BMI: 35 kg/m^2^
			C: nonketogenic very-low-calorie diet	8	44 ± 5	8 (100)	88.2 ± 7.2	37 ± 6		
Westman 2008 [32] (US)	Randomized controlled trial	24 weeks	I: low-carbohydrate, ketogenic diet	38	51.8 ± 7.3	29 (76.3)	178.1 ± 72.9	37.7 ± 6.1	Yes	Age: 18–65 years; BMI: 27–50 kg/m^2^; HbA1c: >6.0%
			C: low-glycemic, reduced-calorie diet	46	51.8 ± 7.8	37 (80.4)	166.8 ± 63.7	38.5 ± 5.6		
Yancy 2007 [33] (US)	Randomized controlled trial (prospective analysis of volunteers from two clinical trials)	24 weeks	I: low carbohydrate ketogenic diet	27	43.8 ± 10.2	19 (70)	87.1 ± 2.2	36.0 ± 4.9	No	Age 18–65 years; BMI: 30–60 kg/m^2^
			C: low fat diet	12	42.8 ± 7.3	9 (75)	95.8 ± 3.1	36.0 ± 5.7		
Yancy 2010 [34] (US)	Randomized controlled trial	48 weeks	I: low-carbohydrate, ketogenic diet	72	52.9 ± 10.2	20 (28)	-	39.9 ± 6.9	No	Age: 18–70 years; BMI: 27–30 kg/m^2^ (plus obesity-related disease) or BMI: ≥30 kg/m^2^
			C: orlistat therapy plus low-fat diet	74	52.0 ± 9.2	21 (28)	-	38.8±7.0		

Abbreviations: SD, standard deviation; FG, fasting glucose; T2DM, type 2 diabetes mellitus; BMI, body mass index; I, intervention; C, control; HbA1c, glycated hemoglobin; OGTT, oral glucose tolerance test.

## Supplementary Materials

The following are available online at https://www.mdpi.com/2072-6643/12/7/2005/s1, Figure S1: Forest plot for association between glycemic control and ketogenic diet in patients with overweight or obesity and with or without T2DM, Figure S2: Forest plots for association between triglyceride control and ketogenic diet in patients with overweight or obesity and with or without T2DM.
