# Supplementary material for: Impact of a Ketogenic Diet on Metabolic Parameters in Patients with Obesity or Overweight and with or without Type 2 Diabetes: A Meta-Analysis of Randomized Controlled Trials
Source: Nutrients. 2020 Jul 6;12(7):2005. doi: 10.3390/nu12072005 (PMC7400909; doi:10.3390/nu12072005)
Supplement: Supplementary file 1 [file nutrients-12-02005-s001.pdf]

Supplementary material

(**a**) Fasting glucose

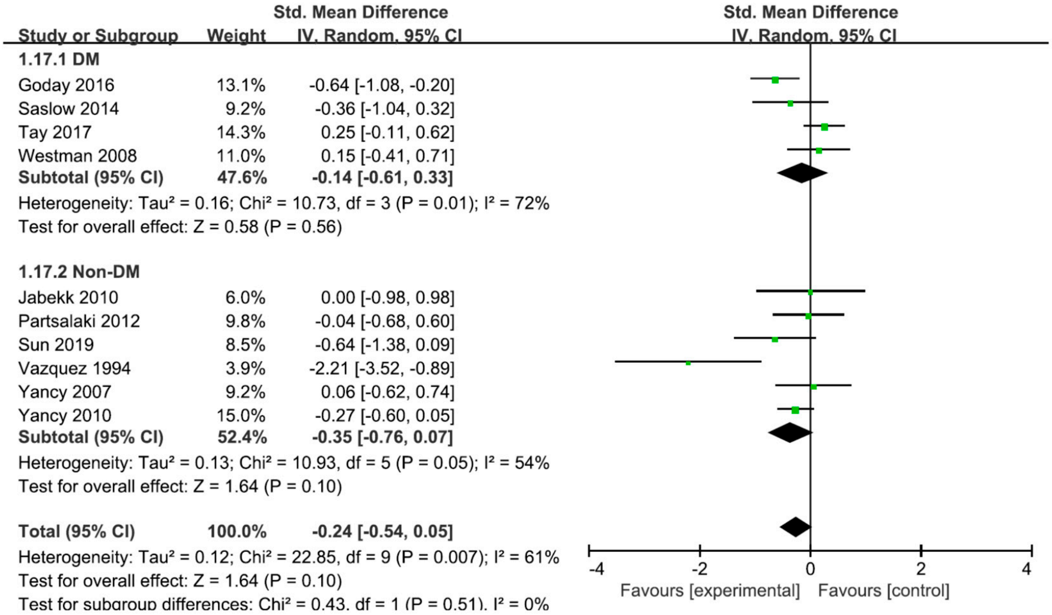

(**b**) Fasting insulin

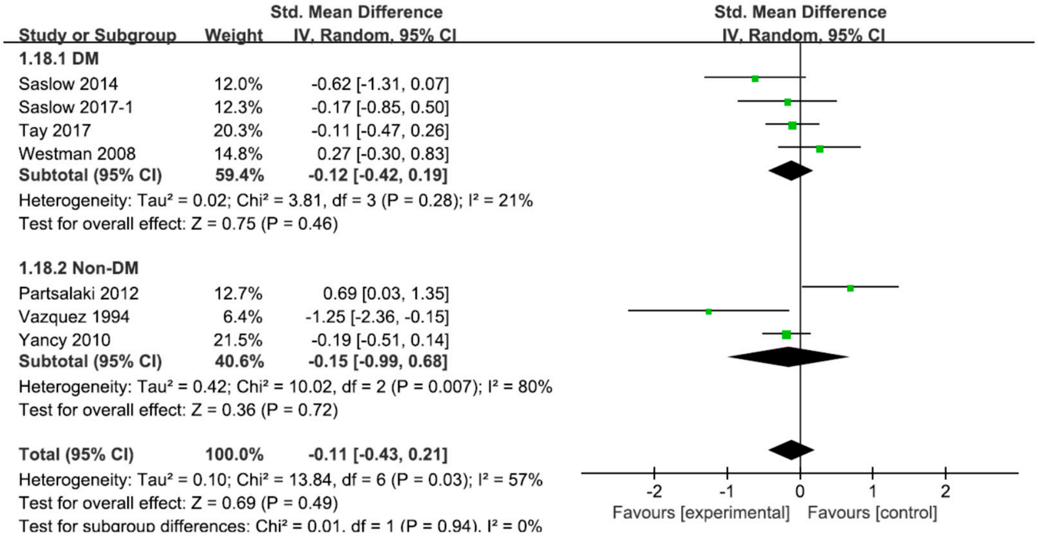

Figure S1. Forest plot for association between glycemic control and ketogenic diet in patients with overweight or obesity and with or without T2DM: (**a**) Changes in fasting glucose; (**b**) Changes in fasting insulin.

Abbreviations: CI, confidence interval; DM, diabetes mellitus; Non-DM, non-diabetes mellitus; T2DM, type 2 diabetes mellitus.

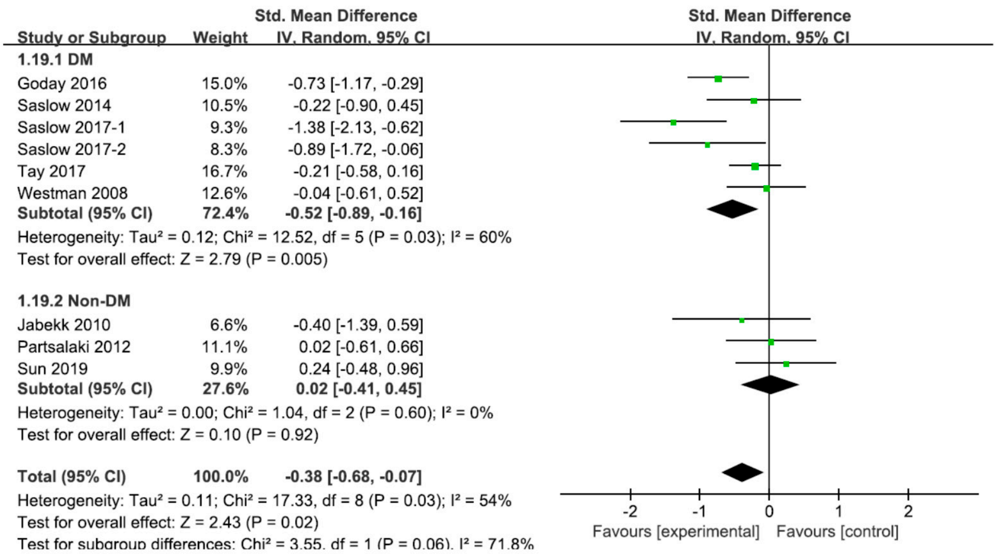

Figure S2. Forest plots for association between triglyceride control and ketogenic diet in patients with overweight or obesity and with or without T2DM.

Abbreviations: CI, confidence interval; DM, diabetes mellitus; Non-DM, non-diabetes mellitus; T2DM, type 2 diabetes mellitus.
